# Reply to Gkegkes et al.

**DOI:** 10.1055/a-2811-5003

**Published:** 2026-02-25

**Authors:** Miguel Fraile-López, Adolfo Parra-Blanco

**Affiliations:** 1 16516 Gastroenterology and Hepatology Department, Clinical and Translational Research in Digestive Diseases, Valdecilla Research Institute (IDIVAL)., Hospital Universitario Marques de Valdecilla, Santander, Spain; 2 9820 NIHR Nottingham Digestive Diseases Biomedical Research Unit, Nottingham University Hospitals NHS Trust, Nottingham, United Kingdom of Great Britain and Northern Ireland



We appreciate the thoughtful comments by Gkegkes Ioannis et al. and their kind recognition of our paper describing the first worldwide experience with endoscopic submucosal dissection (ESD) for treatment of anal squamous cell carcinoma (ASCC). We also thank the editor for the opportunity to clarify several aspects of this approach.

First, en bloc resection of low-grade squamous intraepithelial lesions (LSILs) using ESD enables accurate histopathological evaluation and definitive treatment. Although no studies have specifically evaluated accuracy of optical diagnosis or pre-procedure biopsies in early ASCC, evidence from Western series on esophageal SCC suggests that biopsies may underestimate the final histology of ESD specimens by up to 60% 1 . It is reasonable to assume that similar limitations may apply in the anal canal. Moreover, this approach is particularly advantageous in young patients because it may obviate the need for prolonged or unnecessary surveillance.

In contrast, although ablative therapies have demonstrated efficacy in controlling tumor progression in patients with high-grade squamous intraepithelial lesions (HSIL) - as observed in other anatomical locations in the digestive tract - local excision, when feasible, represents a more definitive diagnostic and therapeutic strategy. Specifically, it allows for R0 resection and is associated with a lower risk of local recurrence 2 . Moreover, in high-volume centers with extensive expertise, rectal ESD has been shown to be safe and feasible in an outpatient setting 3 4 .

Finally, optical diagnosis using flexible endoscopy with chromoendoscopy and magnification, based on intrapapillary capillary loop patterns, has demonstrated excellent diagnostic accuracy for esophageal SCC, reaching up to 90% in Japan 5 . Therefore, in the absence of comparative studies with anoscopy, this technique should be considered a valid and reliable diagnostic tool.

Consequently, ESD should be regarded as a complete excisional biopsy with curative potential. We appreciate these insightful comments and agree that further prospective studies will be essential to advance the understanding and management of this pathology.

**Publication note**

Letters to the editor do not necessarily represent the opinion of the editor or publisher. The editor and publisher reserve the right to not publish letters to the editor, or to publish them abbreviated or in extracts.
